# Evidence-informed guidelines in oral health: insights from a systematic survey

**DOI:** 10.1186/s12903-024-04445-w

**Published:** 2024-06-27

**Authors:** Francisca Verdugo-Paiva, Ana María Rojas-Gómez, Vicente Wielandt, Javiera Peña, Iván Silva-Ruz, Francisco Novillo, Camila Ávila-Oliver, Xavier Bonfill-Cosp, Michael Glick, Alonso Carrasco-Labra

**Affiliations:** 1 Department of Paediatrics, Obstetrics & Gynaecology and Preventive Medicine and Public Health, Universitat Autònoma de Barcelona, Bellaterra (Cerdanyola del Vallès), Barcelona, 08193 Spain; 2 Orofacial Pain & TMD Program, Facultad de Odontologia, Universidad Andres Bello, Room #8, Echaurren 237, Las Condes, Santiago, 8370133 Chile; 3 Epistemonikos Foundation, Mariano Sanchez Fontecilla 368, Las Condes, Santiago, 7550296 Chile; 4 Instituto Craneomandibular, Mestre Nicolau 19, 1, Barcelona, 08021 España; 5 School of Dentistry, Faculty of Medicine, Pontificia Universidad Católica de Chile, Las Condes, Santiago, Chile; 6 Escuela de Odontología, Facultad de Odontología y Ciencias de la Rehabilitación, Universidad San Sebastián, Santiago, Chile; 7 Clinical Epidemiology Service, Hospital Sant Pau, Barcelona, Spain; 8 Iberoamerican Cochrane Centre, Sant Pau Biomedical Research Institute, Barcelona, Spain; 9 Center for Integrative Global Oral Health, School of Dental Medicine, University of Pennsylvania, 240 S 40th. St, Philadelphia, PA 19104 USA

**Keywords:** Guidelines, Guidance, Recommendations, GRADE, Practice statements, Policy, Evidence-to-decision framework, Evidence-based Dentistry, oral Health policies

## Abstract

**Background:**

Oral diseases are a major global public health problem, impacting the quality of life of those affected. While consensus exists on the importance of high-quality, evidence-informed guidelines to inform practice and public health decisions in medicine, appropriate methodologies and standards are not commonly adhered to among producers of oral health guidelines. This study aimed to systematically identify organizations that develop evidence-informed guidelines in oral health globally and survey the methodological process followed to formulate recommendations.

**Methods:**

We searched numerous electronic databases, guideline repositories, and websites of guideline developers, scientific societies, and international organizations (January 2012–October 2023) to identify organizations that develop guidelines addressing any oral health topic and that explicitly declare the inclusion of research evidence in their development. Pairs of reviewers independently evaluated potentially eligible organizations according to predefined selection criteria and extracted data about the organization’s characteristics, key features of their guidelines, and the process followed when formulating formal recommendations. Descriptive statistics were used to analyze and summarize data.

**Results:**

We included 46 organizations that developed evidence-informed guidelines in oral health. The organizations were mainly professional associations and scientific societies (67%), followed by governmental organizations (28%). In total, organizations produced 55 different guideline document types, most of them containing recommendations for clinical practice (77%). Panels were primarily composed of healthcare professionals (87%), followed by research methodologists (40%), policymakers (24%), and patient partners (18%). Most (60%) of the guidelines reported their funding source, but only one out of three (33%) included a conflict of interest (COI) policy management. The methodology used in the 55 guideline document types varied across the organizations, but only 19 (35%) contained formal recommendations. Half (51%) of the guideline documents referred to a methodology handbook, 46% suggested a structured approach or system for rating the certainty of the evidence and the strength of recommendations, and 37% mentioned using a framework to move from evidence to decisions, with the GRADE-EtD being the most widely used (27%).

**Conclusion:**

Our findings underscore the need for alignment and standardization of both terminology and methodologies used in oral health guidelines with current international standards to formulate trustworthy recommendations.

**Supplementary Information:**

The online version contains supplementary material available at 10.1186/s12903-024-04445-w.

## Background

Clinical practice and public health guidelines have evolved as a response to widespread variation in health decision-making at both individual and population levels. Guidelines should contain systematically developed, evidence-informed, actionable statements – known as recommendations – that facilitate patients, clinicians, policymakers, and other relevant stakeholders in making health care and public health policy decisions [1, 2]. The overarching goal of guidelines is not only to improve clinical care and minimize unjustified variations in practice, but also to avoid the use of unnecessary or ineffective services and ensure the effective allocation of healthcare resources [2].

A variety of academic working groups and institutions have developed standards for trustworthy guideline development, such as the U.S. National Academy of Medicine (former Institute of Medicine), the World Health Organization (WHO), the National Institute for Health and Care Excellence (NICE), and the Grading of Recommendations, Assessment, Development, and Evaluation (GRADE) working group [2–5]. Despite nuanced disparities among these standards, a unanimous fundamental accord prevails, which is the systematic identification and synthesis of the available research evidence addressing the potential effects of interventions or options, an exploration of contextual factors (such as the importance of the outcomes, patient values and preferences, resource utilization, stakeholder acceptability, implementation, and equity considerations), an assessment of the certainty of the evidence, the use of a structured process to move from evidence to decisions, and reaching agreement through consensus [1, 5, 6]. In addition, the Assessment of Guidelines for Research and Evaluation (AGREE) tool, a robust guideline appraisal instrument, has emerged as a reliable tool for evaluating methodological quality, offering guideline developers and users standardized means to assess guidelines’ trustworthiness [7].

Organizations worldwide, including scientific societies, professional associations, and Ministries of Health, produce guidelines to support dental practice and inform public oral health decisions. Despite the availability of methodological standards for guideline development in the medical field, oral health guideline developers have not incorporated these criteria, with several guidelines assessed as low quality [8–14]. Furthermore, identifying these documents is challenging, as there is no centralized place to host them, many are not published in peer-reviewed journals, and new updates are rarely disseminated successfully. These concerns lead to a situation where several guidelines on the same topic are available, but intended users, such as clinicians, policymakers, hospital administrators, and other stakeholders, struggle to find these documents and decide which to use to inform their decisions.

Trustworthy guidelines developed with rigorous methods are essential to translate evidence into clinical practice and policy, but developing them requires substantial resources (time, training, panel meetings, software support, evidence synthesis deliverables, publication and dissemination platforms, and implementation strategies). Adopting existing recommendations from guidelines developed previously by other organizations or adapting them to another context may efficient alternatives, especially in low- and middle-income settings [15]. Proper adoption or adaptation of guidelines requires a rigorous description of the processes used by the developers of the original guideline, but existing guidelines often do not provide the necessary information to facilitate their adaptation or adoption [2, 15].

The WHO and the FDI World Dental Federation (FDI) have been calling for urgent improvements in oral health systems, highlighting the importance of translating research findings into practice, including developing region-specific, evidence-informed guidelines [16, 17]. However, there is still a lack of a collective problem-solving orientation to leverage evidence for decision-making [18], with a lack of coordination and dialogue among different stakeholders across the oral health evidence ecosystem.

To accelerate the development and availability of high-quality, evidence-informed oral health guidelines, this systematic survey aimed to identify organizations worldwide that develop evidence-informed guidelines in oral health and provide a comprehensive overview of these organizations’ characteristics and methods applied when formulating recommendations.

## Methods

We published our systematic survey protocol [19] and presented the findings using previously published systematic survey reporting formats as a model [20].

### Eligibility criteria

We included organizations that develop evidence-informed guidelines in oral health. We considered a ‘guideline’ any document or information product containing actionable statements recommending or suggesting a course of action to guide clinical practice or public health-related decision-making [2, 4], regardless of the terminology to refer to these documents (e.g., clinical practice guideline, guidance, consensus guideline, consensus recommendation). In addition, the organization must have produced at least three guidelines in the past ten years, regardless of the oral health topic, according to the oral health definition provided by the FDI and WHO [21, 22]. ​​We decided to use this threshold as an eligibility criterion because we are interested in describing the methodological process followed by organizations dedicated to guideline development. We wanted to distinguish them from organizations that sporadically produced guidelines to avoid including organizations that produced guidelines one time or for particular reasons (e.g., organizations that produced guidelines for dental care during COVID-19 pandemic only).

Organizations producing guidelines but not explicitly declaring the inclusion of research evidence for their development were excluded. We also excluded organizations that solely produce educational or health system policy documents.

### Information sources and selection of organizations

We conducted a systematic search in MEDLINE, Epistemonikos, and guideline repositories (i.e., CPG Infobase, the International Guidelines Library from GIN, the Guideline Central, the Alliance for the Implementation of Clinical Practice Guidelines, and Medical Information Distribution Service Guidelines Library) from January 2012 to October 2023, with no language or publication status restrictions. The Epistemonikos database covers more than ten sources of biomedical literature, including but not limited to the Cochrane Database of Systematic Reviews, Pubmed/MEDLINE, EMBASE, CINAHL, PsycINFO, LILACS, DARE, Campbell Library, JBI Database of Systematic Reviews and Implementation Reports and EPPI-Centre Evidence Library [23]. We manually screened the websites of guideline developers, scientific societies, professional associations, and Ministries of Health. The search strategy and sources used are listed in the supplementary material (Appendix 1).

After achieving optimal calibration, pairs of reviewers independently evaluated citations and documents’ initial eligibility and extracted the names of the responsible organizations for the guideline included in the report’s full text to create an initial database. In a second stage, pairs of reviewers independently evaluated whether the organizations previously identified from the systematic and the manual searches were eligible, according to predefined criteria outlined above. We also examined the reference lists of eligible documents and consulted with experts in the guideline development field to identify any missing organizations.

### Data collection

Two calibrated reviewers independently extracted data. Disagreements were solved through discussion and consensus or with the help of a third reviewer. The following data were extracted:

- Organization: We classified the organization type (non-governmental organization, governmental organization, or academic and research institution), country, language, oral health clinical specialty [24, 25], number of documents produced over the last 10 years, and the types of documents produced (documents containing clinical practice or public health-related actionable statements).
- Guidelines: For each document type produced by a single organization, we extracted information about the characteristics of the guideline document and its methodological features, including intended users, stakeholders’ involvement, working group or panel composition, conflicts of interest (COI) management policy, and funding source. For example, if an organization produces more than one document type with a different methodology (for example, a Ministry of Health produces clinical practice guidelines and policy statements), we extracted the data for each document type independently. We reviewed and classified every actionable statement reported in the guideline to determine whether they contain formal recommendations according to a pre-defined taxonomy [26]. A formal recommendation should be the result of a structured process, and they should be explicitly linked to the underpinning evidence resulting from a systematic literature search and appraisal process [26]. A structured process should be followed, including a description of the domains, factors, or criteria considered, supporting panels or stakeholders to move from the available evidence to a recommendation [27].
- Actionable statements: We extracted information related to the formal recommendation development process reported in the guideline, including the type of methodological handbook used (e.g., international organization handbook, in-house handbook), methods for searching and identifying research evidence to inform the guideline (e.g., systematic review, previous guidelines), and information about the process and framework for moving from the evidence to the decisions and formulation of recommendations (e.g., the GRADE-Evidence-to-Decision framework).

The information from each organization, the document type, and the formal recommendation development processes were collected from various sources, such as the organization’s official websites, guideline methods section, reference manuals, or methodological handbooks. If an organization has changed the methods for developing formal recommendations over the years, we extracted the data from the latest published guideline or methodological handbook.

### Data analysis

Descriptive statistics, including mean and median and their corresponding measures of dispersion, were used to describe the data. Absolute frequencies and proportions were calculated for all variables. In addition, we identified taxonomies to classify, for example, how the organizations describe their methods to assess the certainty of the evidence, determine the direction and strength of actionable statements, and use frameworks to move from the evidence to the decisions. In an iterative process, we reviewed and updated these taxonomies as new categories emerged.

## Results

### Search results

**Fig. 1:**
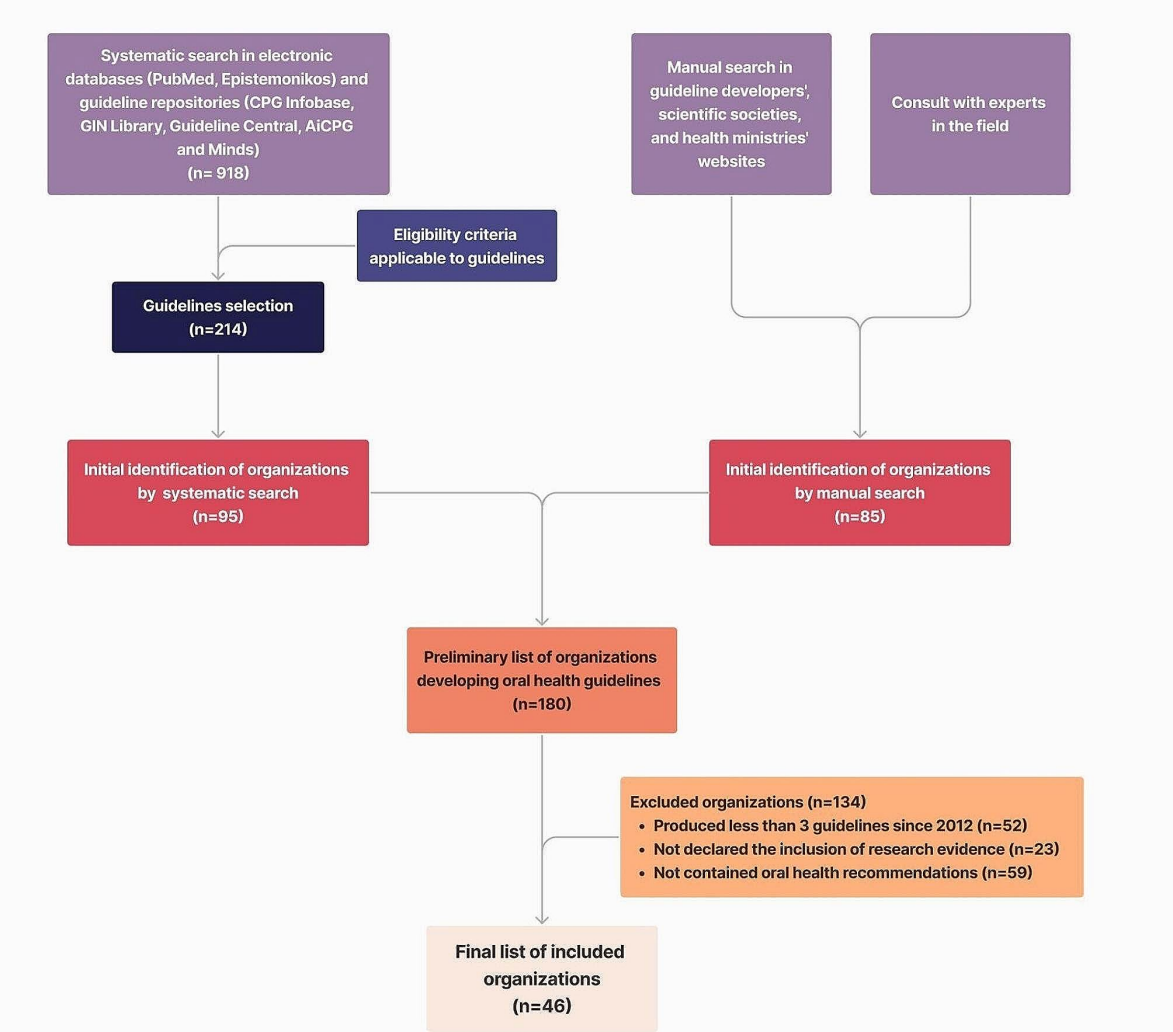
Selection process flowchart CPG: Clinical Practice Guidelines; GIN: Guideline International Network; AiCGP: the Alliance for the Implementation of Clinical Practice Guidelines; Minds: Medical Information Distribution Service Guidelines Library

After removing duplicates, the systematic search on electronic databases retrieved 918 hits, which were screened using title and abstract. We identified 214 guideline documents published by 95 distinct guideline developer organizations as potentially eligible. Our manual search identified 85 potentially eligible guideline developer organizations. In total, we deemed 180 guideline development organizations eligible, of which 46 were included (Fig. 1, Appendix 2).

### Characteristics of included organizations

**Fig. 2:**
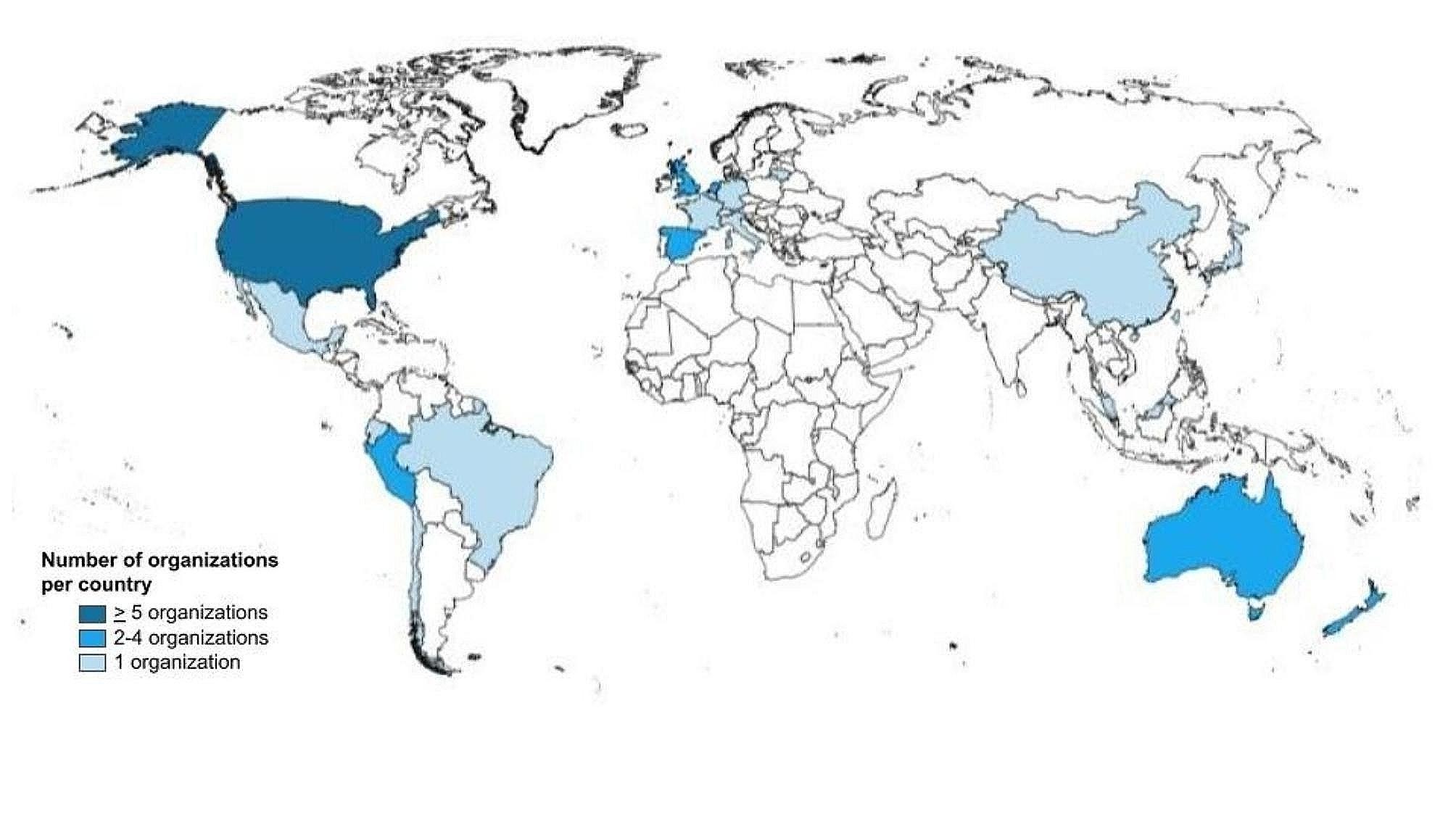
Geographical distribution of organizations producing oral health guidelines worldwide. *This figure does not show four global, six regional European, and one Latin American organization

The identified organizations (*n* = 46) were mainly non-governmental organizations, such as professional associations and scientific societies (67%), followed by governmental organizations (28%) and academic institutions or research groups (5%) (Table 1). Nearly half of the organizations were located in Europe (41%), followed by North America (22%) and South America (13%). The country with the largest number of organizations was the United States (*n* = 9, 20%), followed by the Netherlands, which had three organizations (6%) (Fig. 2). Half of the organizations published their guidelines in English (50%), followed by eight in Spanish (17%).

**Table 1:** General characteristics of organizations developing oral health guidelines worldwide

General characteristics	Organizations (*n* = 46)	
	*n*	%
**Organization type**		
Non-governmental organizations (Scientific society or professional association)	31	67%
Governmental organization (Ministry of Health or governmental healthcare agency)	13	28%
Academic and research institutions (University, faculty, research center)	2	5%
Continent*		
Europe	19	41%
North America	10	22%
South America	6	13%
Oceania	4	9%
Asia	3	7%
Africa	0	0%
**Language**		
English	23	50%
Other (Dutch, French, Mandarin Chinese, German, Italian etc.)	9	20%
Spanish	8	17%
More than one language	6	13%
Dental clinical specialty**		
More than one specialty	19	41%
Pediatric Dentistry	5	11%
Dental Public Health	4	9%
Oral and Maxillofacial Surgery	3	7%
Periodontics	3	7%
Restorative dentistry	3	7%
General dentistry	3	7%
Orofacial Pain	2	5%
Endodontics	1	2%
Oral medicine	1	2%
Prosthodontics	1	2%
**Number of guidelines produced per organization since 2012**		
3 to 5	23	50%
6 to 10	14	30%
*≥* 11	9	20%

*This category does not include four additional global organizations: FDI World Dental Federation (FDI), International Association of Dental Traumatology (IADT), International Caries Consensus Collaboration (ICCC) and the International Association of Paediatric Dentistry (IAPD)

**Dental clinical specialties were classified according to the list and definition of recognized dental specialties approved by the National Commission for the Recognition of Dental Specialties and Certification Boards of the American Dental Association (ADA) and European recognized dental specialties [25, 26]

Most organizations developed guidelines addressing topics across multiple dental specialties (41%). Pediatric dentistry (11%) was the most common dental specialty for which organizations developed guidelines, followed by dental public health (9%). We did not identify any organization that regularly develops guidelines on special care dentistry, dental anesthesiology, oral and maxillofacial radiology, and oral and maxillofacial pathology.

### Characteristics of guideline documents from included organizations

​​The 46 included organizations produced 55 different guideline documents. Most consistently produced only one document type containing oral health recommendations (87%), and only six institutions (13%) produced more than one document type. For example, the German Society for Dental, Oral and Maxillofacial Medicine, coordinated by the Association of the Scientific Medical Societies in Germany, produced four different document typescontaining recommendations for clinical practice: S1 - Expert recommendation; S2e - Evidence-based guideline; S2k Consensus-based guidelines, and S3 - Evidence- and consensus-based guideline. Each document type was evaluated separately since each one has its own characteristics and follows a different methodology.

In addition, the majority (77%) of the 55 guideline documents addressed recommendations for oral health care only, while a smaller percentage (13%) of documents contained oral public health-related recommendations, followed by documents containing both oral health care and oral public health-related recommendations (10%). For example, the Italian Ministry of Health produces national guidelines, with a document providing recommendations for the primary prevention of dental trauma at home and school and oral health care recommendations for managing dental trauma [28].

The scope of implementation varied across the guidelines documents, with the majority (80%) of the 55 guideline documents written for national-level implementation, followed by global (11%) and regional-level implementation (7%).

Although most (62%) of the 55 guidelines documents reported that the intended users of the documents were healthcare professionals, a considerable percentage reported other multistakeholder groups as their users (e.g., clinicians, patients, students, and policymakers) (25%). Seven (13%) guideline document types did not report the intended users of their guidelines. Most of the guideline documents provided some information about the composition of the panel involved in the guideline development process. These panels were primarily composed of healthcare professionals (87%), with a much smaller percentage (40%) reporting the involvement of research methodologists (researchers with expertise in evidence synthesis and guidelines methodology), policymakers (24%), and patient partners (18%). Six (11%) guideline documents produced by five organizations did not report any information about the panel involved in the guideline development.

**Fig. 3:**
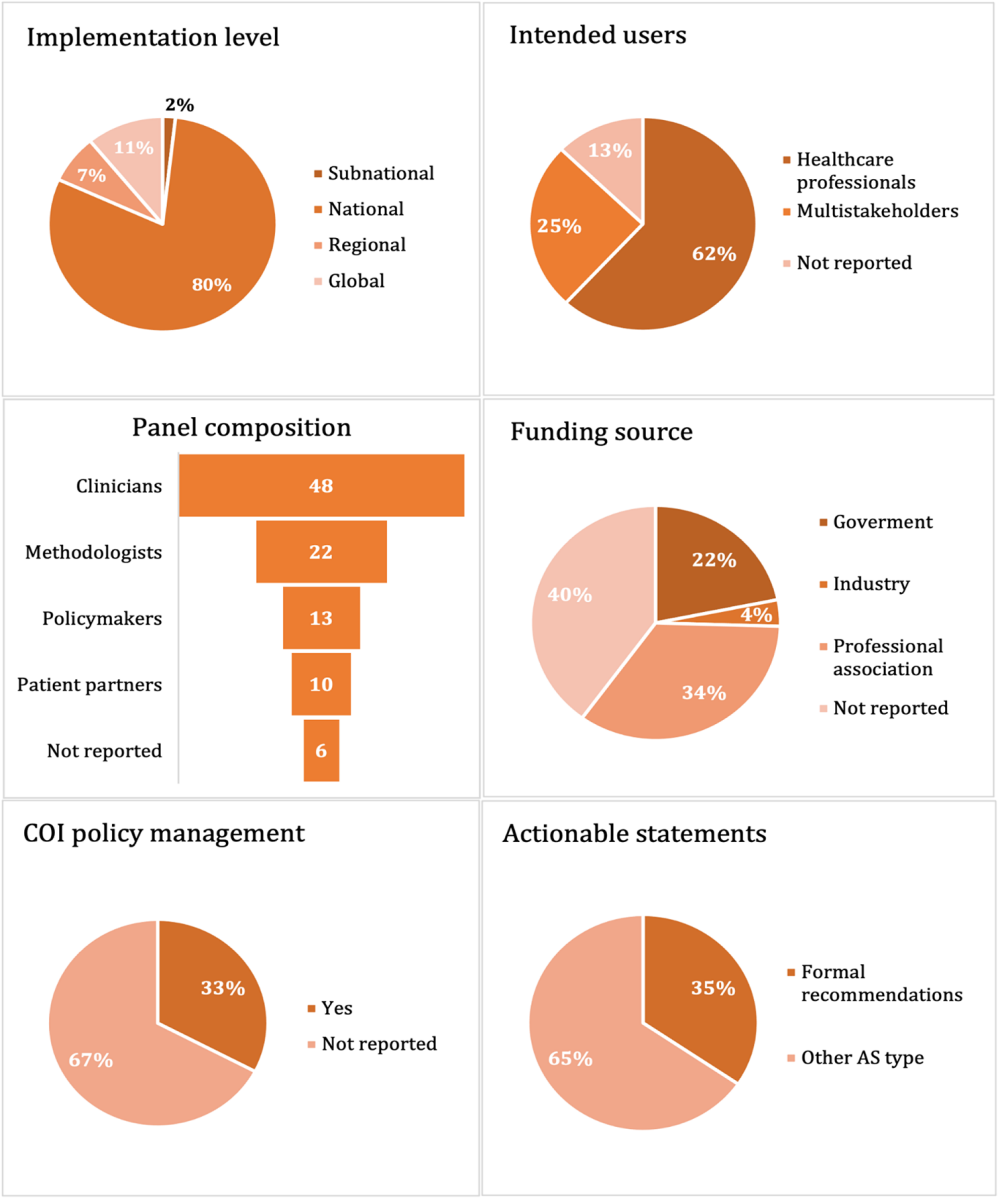
Characteristics of guideline document types produced by oral health organizations (*n* = 55)

Regarding COI, although most guidelines included brief information about the panel’s disclosure of COI, one out of three (33%) documents described a COI policy management to protect guideline integrity. Two out of three (60%) guideline documents disclosed the source of funding for development (Fig. 3 and Appendix 3).

We observed extensive inconsistency in the terminology used by the different organizations to describe the type of guideline document they produce. Guideline developer organizations use more than 20 different terms to refer to documents that contained recommendations to inform oral health care, and eight terms in documents that provided oral public health-related recommendations (Table 2 and Appendix 2).

**Table 2:** Terminology used by oral health organizations to describe the type of document produced

Documents containing oral health care recommendations	Documents containing oral public health-related recommendations
Advice Best clinical practice guidance Best evidence consensus statement Best practice guideline Clinical guidelines Clinical practice guidelines Clinical practice recommendations Clinical practice statements Clinical report Consensus document Consensus recommendations Consensus statements Consensus-based guidelines Evidence- and consensus-based guideline Evidence-based guideline Expert recommendation Good clinical practice Guidance Guidelines Position statements Recommendations Standards	Guidelines Guidance Policy statements Position statements Protocols Recommendation statements Statements Quality standards

### Characteristics of recommendations contained in guidelines documents

The methodology for developing recommendations from the 55 included guidelines showed large variability across organizations, but only 19 of the 55 guidelines documents (35%) contained formal recommendations (Fig. 3). The other 65% of guideline documents contained recommendations where the methods for searching, assessing the certainty of evidence, or evaluating the strength of recommendations were not reported.

Half (51%) of the guideline documents referred to a methodology handbook, including an in-house handbook (24%), the use of a specific methodology or evaluation resources (e.g., GRADE, AGREE II) (16%), or national organization handbooks (11%). Although most (60%) of the 55 guidelines documents reported conducting evidence searches in at least one electronic database, a considerable percentage did not report any information about their methods for searching and identifying research evidence (40%). One out of three (31%) guideline documents mentioned conducting searches to retrieve previously published guidelines.

Almost half (46%) of the guidelines suggested a structured approach or system for rating the certainty (also referred to as “quality”) of the evidence and the strength of recommendations, with the GRADE approach being the most widely used (29%). Still, this information was not reported for most of the guidelines (54%). The GRADE-EtD framework was the most frequently reported approach for moving from evidence to decisions (27%). However, the information about the framework used to move from evidence to decisions was unclear for a considerable number of guidelines (63%) (Table 3).

**Table 3:** Features of the process to produce recommendations in the included guideline documents

Development methods	Formal recommendations from guideline documents (*n* = 19)		All recommendations from guideline documents (*n* = 55)	
	*n*	%	*n*	%
**Use of a handbook**				
Not reported	0	0	27	49%
In-house handbook	7	37%	13	24%
Guideline development methodology (e.g., GRADE, AGREE II)	7	37%	9	16%
National organization handbook (e.g., MoH, ADA, USPSTF)	5	26%	6	11%
Global organization handbook (e.g., WHO)	0	0%	0	0%
**Methods for searching research evidence**				
Search in at least one electronic database	19	100%	33	60%
Not reported	N/A	N/A	22	40%
**Search for previous guidelines**	12	63%	17	31%
**Methodology to assess the certainty/quality of evidence**				
Not reported	N/A	N/A	30	54%
GRADE	14	74%	16	29%
Other	1	6%	4	7%
Own approach	2	10%	2	4%
USPSTF	2	10%	2	4%
SIGN	0	0%	1	2%
**Approach for grading the strength of recommendations**				
Not reported	N/A	N/A	30	54%
GRADE	14	74%	16	29%
Other	2	10%	5	9%
Own approach	2	10%	2	4%
USPSTF	1	6%	1	2%
SIGN	0	0%	1	2%
**Frameworks used to move from evidence to decisions**				
Not reported	N/A	N/A	35	63%
GRADE-EtD	15	78%	15	27%
Own approach	2	10%	2	4%
Other	1	6%	2	4%
USPSTF	1	6%	1	2%

GRADE: Grading of Recommendations, Assessment, Development, and Evaluation; AGREE II: Appraisal of Guidelines, Research and Evaluation; MoH: Ministry of Health; ADA: American Dental Association; USPSTF: U.S. Preventive Services Task Force; WHO: World Health Organization; SIGN: Scottish Intercollegiate Guidelines Network; EtD: Evidence to Decision; N/A: Not applicable.

## Discussion

### Main findings

This study presents the first comprehensive and systematic evaluation of organizations developing evidence-informed guidelines in oral health. Our study reveals an unequal geographical distribution of the organizations worldwide. For example, we did not identify any African guideline organization and found a limited number of organizations and guidelines using a global and regional scope. Half of all oral health guideline development organizations reside in the United States or European countries and use a national-level scope.

Our analyses also show a complete lack of consensus regarding the terminology the guidelines organizations use to describe the type of document they produce, with more than twenty variations to refer to guidelines informing oral health care and eight for oral public health-related documents. This inconsistency in terminology threatens the usability and application of formal recommendations by target users and limits the ability of oral health guidelines to transcend to other health care professionals, policymakers, and patients.

Another alarming finding is the small percentage of organizations involving different stakeholder groups in their guideline development process. Most organizations only produce guidelines by and for clinicians. Two-thirds of the organizations did not have a COI policy management, with more than 40% not providing information about funding sources to support guideline development.

Finally, our study shows that only one-third of the organizations conduct a structured process for formulating formal recommendations. While oral health guidelines providing recommendations claim using international standards, the methods used are inconsistent, informal, and deviate from standards. For example, many developers reported using in-house handbooks and applying their own approach to guideline development.

### Our results in the context of previous research

Previous research in Medicine shows that GRADE is the most widely used approach to assess the certainty of the evidence and grade the strengths of recommendations in healthcare guidelines [29–31]. Our study findings aligned with these results since the GRADE approach was the most frequently used methodology by organizations developing formal recommendations. However, the proportion of oral healthcare organizations developing guidelines containing statements that result from a structured process is low. In a recently published methodological study, Meneses-Echavez et al. reported that most healthcare organizations in Medicine used a system for grading the strength of recommendations (88%), and two out of three guideline documents (66%) have a framework to guide the EtD process [31]. In our study, only 46% of the guideline document types reported a system for grading the strength of recommendations, and 37% mentioned using a framework to move from evidence to decisions. The same study reported that more than half of the documents (64%) mentioned the involvement of patient partners in guideline panels (32, while in our study, only 18% of the guideline documents mentioned the involvement of patient representatives. The same pattern was observed for the management of COI description, with 59% and 32%, respectively [31].

Our findings confirm substantial methodological deficiencies across oral health guidelines, highlighting the limited adherence to current international standards to formulate trustworthy recommendations. This situation has been previously reported in several studies [8–14]. A recently published systematic survey evaluated the methodological quality of guidelines addressing the management of traumatic dental injuries. The authors found that a small percentage of the included guidelines provided any information about their methodology, with only one guideline using the GRADE approach [13].

Another major weakness among oral health organizations producing guideline documents is the inconsistent and outdated terminology in the guideline development process. For example, although several organizations mentioned including research evidence to develop their documents, many still distinguish between evidence-based and consensus-based guidelines, which is misguided and misleading [32]. All clinical practice and public health guideline recommendations require careful consideration of the relevant evidence and consensus from the development group, independently of the certainty of the evidence available. Guidelines that do not use systematic methods to summarize the best available evidence to inform a consensus process of formulating formal recommendations are simply non-evidence-based.

### Limitations and strengths

Our study has some limitations. Although we performed a comprehensive search, we may have missed relevant oral healthcare organizations. This is expected since several organizations don’t publish their guidelines in academic journals, so the only way to capture them is by manually searching their websites. Nevertheless, our manual searches covered a representative number of organizations’ websites worldwide, including more than fifty ministries of health websites. Likewise, the data extraction process was not exempt from challenges. Since the terminology and the methods for the guideline development were inconsistent across the organizations, the data were extracted and analyzed through iterative consensus between researchers. To avoid error and bias, we followed methodological standards for conducting evidence synthesis studies, such as piloting the data extraction before starting and conducting a duplicate screening and data extraction process. We did not analyze the guidelines’ content, the strength, direction, or wording of the recommendations. Likewise, it is not possible to conclude that there are meaningful differences between recommendations formulated following a structured or an unstructured process. However, final recommendations depend on several factors, such as the scientific evidence used, the methodological process, stakeholders’ involvement, panel decisions, and other contextual factors (e.g., available resources, patients’ values and preferences, and implementation issues). Differences between recommendations formulated by two organizations on the same topic can arise for multiple reasons.

Our study also has several strengths, including our comprehensive search, the detailed work identifying organizations’ research methods beyond the guideline document itself (e.g., handbooks), and the use of an iterative and systematic approach to data extraction.

### Implications for practice and research

The number of available guidelines in oral health has grown substantially during the last few years. However, there is still a need for substantial improvement in the quality and trustworthiness of those guidelines. Although several guidelines are available for the most relevant oral health topics worldwide, only a few can be recommended for use in practice and rated as high quality [11–13]. As the integration of oral health into universal health coverage gains global priority, closely aligning with broader health and well-being agendas, translating and implementing research evidence through trustworthy evidence-informed guidelines becomes imperative. This is crucial, for example, to achieve comprehensive integration of medical and dental care. Oral health guidelines must adopt accepted international methodological standards already available in the medical field.

Evidence-informed guidelines improve clinical practice by promoting interventions of proved benefit, discouraging ineffective ones, and improving the consistency of care [33, 34]. However, developing evidence-informed guidelines is not enough since their impact depends on how the implementation stage is carried out. Guidelines must reach the clinicians who will use them, and these practitioners must understand them [35]. Organizations developing guidelines must ensure the recommendations are feasible, actionable, operational, and acceptable to all stakeholder groups to achieve optimal adherence. Additionally, well-designed dissemination strategies are required [35]. Studies suggest that evidence-based recommendations are better followed in practice than recommendations not based on scientific evidence [36, 37], but further research is still needed on the effect of high-quality, evidence-informed guidelines in oral health.

High-quality guidelines, especially those produced with a global or regional scope of implementation, can facilitate the rapid adoption or adaptation of recommendations in low and middle-income countries. These efficiencies allow organizations to save time and resources by reusing up-to-date data generated from previous guidelines to develop contextualized recommendations. Successful examples of guideline adaptation for caries management in Iranian and Malaysian populations have been published recently [38, 39]. Nevertheless, even adopting or adapting guidelines requires a rigorous description of the processes used by the source guideline [15].

Given the pivotal importance of evidence-informed guidelines in enhancing both oral and general health, the results of this study underscore the need for alignment and standardization of both terminology and methodologies used in oral health with those recognized internationally in the medical field. Implementing and continuously improving those methods would require establishing worldwide collaborative networks dedicated to oral health guidelines, which have the potential to significantly change the current situation. These networks would aid organizations in adhering to contemporary methodological norms, prevent redundant efforts, and ensure easier access to top-tier guidelines, digital technologies, and methodological innovations. However, these improvements may prove insufficient. Extensive stakeholder engagement initiatives connecting policymakers, healthcare agencies, patient partners, and clinicians will allow guideline developers to prioritize topics and produce recommendations readily available for implementation, adoption, or adaptation globally.

## Conclusion

Our findings suggest a lack of consensus regarding the terminology guidelines organizations use, along with inconsistent and informal methods for the guidelines development process in oral health guidelines globally. There is an urgent need to standardize and elevate the methodological quality of oral health guidelines worldwide. Such a standard requires a rigorous methodology to synthesize the available evidence, a structured process to move from the available evidence to recommendations, independent funding sources, COI policy management, and a multidisciplinary working group involvement [2, 4, 27].

### Electronic supplementary material

Below is the link to the electronic supplementary material.

Supplementary Material 1

Supplementary Material 2

Supplementary Material 3

## Data Availability

The data that support the findings of this study are available from the corresponding author upon request. All relevant data from the study are included in the figures and tables in the manuscript and supplementary material.

## Declarations

### Study registration

The protocol of this systematic survey can be found in: Verdugo-Paiva F, Bonfill Cosp X, Alonso-Coello P, Ávila-Oliver C, Glick M, Carrasco-Labra A. How oral health care organizations formulate actionable statements to inform practice and policy: A protocol for a systematic survey. F1000Res. 2023;12:1261. Published 2023 Oct 3. doi:10.12688/f1000research.141423.1.

## Abbreviations

WHO: World health organization
NICE: National institute for health and care excellence
GRADE: Grading of recommendations, assessment, development, and evaluation
COI: Conflict of Interest
FDI: FDI world dental federation
ADA: American dental association
AGREE II: Appraisal of guidelines, research and evaluation
MoH: Ministry of health
USPSTF: U.S. preventive services task force
SIGN: Scottish intercollegiate guidelines network
EtD: Evidence to Decision
