# Supplementary material for: Evidence-informed guidelines in oral health: insights from a systematic survey
Source: BMC Oral Health. 2024 Jun 27;24:746. doi: 10.1186/s12903-024-04445-w (PMC11212404; doi:10.1186/s12903-024-04445-w)
Supplement: Supplementary file 1 — Supplementary Material 1 [file 12903_2024_4445_MOESM1_ESM.docx]

**Appendix 1. Search strategy and sources**

| **Database** | **Search strategy** | **Hits** |
| --- | --- | --- |
| PubMed | (dentist* OR dental* OR dentin* OR dentate* OR dentition* OR teeth* OR tooth* OR odonto* OR molar* OR maxilla* OR maxillo* OR maxille* OR mandib* OR jaw OR temporomandib* OR "temporo-mandibular" OR "temporo mandibular" OR tmj OR tmd OR craniomandib* OR "cranio-mandibular" OR "cranio mandibular" OR craniomaxillo* OR craniofacial* OR "cranio-facial" OR "cranio facial" OR prosthodont* OR edentul* OR exodont* OR "oral health" OR "oral care" OR "oral hygiene" OR "oral cancer" OR "oral surgery" OR orofacial* OR "oro-facial" OR "oro facial" OR gingivit* OR periodont* OR endodontic* OR "root canal" OR ocluss* OR orthodont* OR caries* OR carious* OR mouth* OR bruxism* OR dentur* OR cariology* OR teledentist* OR gerodontology) AND (guideline* OR guidance* OR recommendation* OR consensus* OR statement* OR policy* OR policies* OR "Health Planning Guidelines"[Mesh] OR "Consensus"[Mesh] OR "Guidelines as Topic"[Mesh]) | 908 |
| Epistemonikos database | (dentist* OR dental* OR dentin* OR dentate* OR dentition* OR teeth* OR tooth* OR odonto* OR molar* OR maxilla* OR maxillo* OR maxille* OR mandib* OR jaw OR temporomandib* OR "temporo-mandibular" OR "temporo mandibular" OR tmj OR tmd OR craniomandib* OR "cranio-mandibular" OR "cranio mandibular" OR craniomaxillo* OR craniofacial* OR "cranio-facial" OR "cranio facial” OR prosthodont* OR edentul* OR exodont* OR “oral health” OR “oral care” OR “oral hygiene” OR “oral cancer” OR “oral surgery” OR orofacial* OR "oro-facial" OR "oro facial" OR gingivit* OR periodont* OR endodontic* OR “root canal” OR ocluss* OR orthodont* OR caries* OR carious* OR mouth* OR bruxism* OR dentur* OR cariology* OR teledentist* OR gerodontology) AND (guideline* OR guidance* OR recommendation* OR consensus* OR statement* OR policy* OR policies*) | 203 |

**List of sources and websites for manual search.

Guidelines repositories**

| **Name** | **URL** |
| --- | --- |
| CPG Infobase: Clinical Practice Guidelines | <https://joulecma.ca/cpg/homepage> |
| International Guidelines Library - Guideline International Networks (GIN) | <https://guidelines.ebmportal.com/> |
| Guideline Central | <https://www.guidelinecentral.com/> |
| The Alliance for the Implementation of Clinical Practice Guidelines (AiCPG) | <https://aicpg.org/> |
| Medical Information Distribution Service (Minds) - Japan Council for Quality Health Care. | <https://minds.jcqhc.or.jp/english> |

**Guideline developers', scientific societies and international organizations websites**

| **Name** | **URL** |
| --- | --- |
| National Institute for Health and Care Excellence | <http://www.nice.org.uk> |
| Scottish Intercollegiate Guidelines Network | <http://www.sign.ac.uk> |
| Guía Salud | <https://portal.guiasalud.es> |
| Australian Clinical Practice Guideline | <https://www.clinicalguidelines.gov.au> |
| Scottish Dental Clinical Effectiveness Programme | <http://www.sdcep.org.uk> |
| IECS: Instituto de Efectividad Clínica y Sanitaria | <https://www.iecs.org.ar> |
| Agency for Healthcare Research and Quality (AHRQ) | <https://www.ahrq.gov/research/findings/evidence-based-reports/search.html> |
| Scottish dental | <https://www.scottishdental.org/professionals/guidelines/> |
| World Health Organization (WHO) | <https://www.who.int/es> |
| Organización Panamericana de la Salud (OPS) | <https://www.paho.org/en> |
| American Dental Association (ADA) | <https://www.ada.org/en> |
| FDI World Dental Association | <https://www.fdiworlddental.org> |
| International Association for Dental Research (IADR) | <https://www.iadr.org> |
| American Academy of Pediatric Dentistry (AAPD) | <http://www.aapd.org/> |
| Australian and New Zealand Society of Pediatric Dentistry (ANZSPD) | <https://www.anzspd.org.au/> |
| European Academy of Paediatric Dentistry (EAPD) | <http://www.eapd.gr/> |
| International Association of Paediatric Dentistry (IAPD) | <http://www.iapdworld.org/> |
| Pediatric Dentistry Association of Asia (PDAA) | <http://pdaasia.org/> |
| California Dental Association (CDA) | <https://www.cda.org/> |
| ICDAS Foundation | https://www.iccms-web.com/ |
| General Dental Council (GDC) | https://www.gdc-uk.org/ |

**Ministries of Health websites**

| **Country** | **URL** |
| --- | --- |
| Nigeria | <http://www.fmh.gov.ng> |
| South Africa | <http://www.doh.gov.za> |
| Sudan | <http://www.fmoh.gov.sd> |
| Tanzania | <http://www.moh.go.tz> |
| Uganda | <http://health.go.ug/mohweb> |
| Argentina | <https://www.argentina.gob.ar/salud> |
| Brasil | <https://saude.gov.br> |
| Bolivia | <https://www.minsalud.gob.bo> |
| Chile | <http://www.minsal.cl> |
| Colombia | <https://www.minsalud.gov.co/portada-covid-19.html> |
| Costa Rica | <http://www.ministeriodesalud.go.cr> |
| Cuba | <http://www.sld.cu> |
| Dominican Republic | <http://www.salud.gob.do> |
| El Salvador | <http://www.salud.gob.sv> |
| Nicaragua | <http://www.minsa.gob.ni> |
| Perú | <https://www.gob.pe/minsa> |
| Trinidad and Tobago | <http://www.health.gov.tt> |
| United States of America | <http://www.hhs.gov> |
| Bhutan | <http://www.health.gov.bt> |
| Cambodia | <http://www.moh.gov.kh> |
| China | <http://www.moh.gov.cn> |
| Indonesia | <http://www.depkes.go.id> |
| Iraq | <http://www.moh.gov.iq> |
| Japan | <http://www.mhlw.go.jp> |
| Nepal | <http://www.mohp.gov.np> |
| Pakistan | <http://www.pakistan.gov.pk> |
| Singapore | <http://www.health.gov.lk> |
| Thailand | <http://eng.moph.go.th> |
| Turkey | https://www.saglik.gov.tr/ |
| Austria | <http://www.bmg.gv.at> |
| Belgium | <http://www.health.belgium.be> |
| Cyprus | <http://www.moh.gov.cy> |
| Denmark | <http://www.sst.dk> |
| England | https://www.gov.uk/government/organisations/public-health-england |
| France | <http://www.sante.gouv.fr> |
| Germany | <http://www.bmg.bund.de> |
| Greece | <http://www.yyka.gov.gr> |
| Iceland | <http://www.velferdarraduneyti.is> |
| Ireland | <http://www.dohc.ie> |
| Italy | <http://www.salute.gov.it> |
| Netherlands | <http://www.government.nl/ministries/vws> |
| Norway | <http://www.regjeringen.no> |
| Portugal | <http://www.portaldasaude.pt> |
| Scotland | <http://www.healthscotland.scot/> |
| Spain | <http://www.msc.es> |
| Singapore | <https://www.moh.gov.sg/hpp/dentists/guidelines> |
| Sweden | <http://www.folktandvardenstockholm.se> |
| Switzerland | <http://www.bag.admin.ch> |
| Australia | <http://www.health.gov.au> |
| New Zealand | <http://www.health.govt.nz> |
