# Supplementary material for: Evidence-informed guidelines in oral health: insights from a systematic survey
Source: BMC Oral Health. 2024 Jun 27;24:746. doi: 10.1186/s12903-024-04445-w (PMC11212404; doi:10.1186/s12903-024-04445-w)
Supplement: Supplementary file 2 — Supplementary Material 2 [file 12903_2024_4445_MOESM2_ESM.pdf]

Appendix 2. Included organizations developing oral health guidelines

Organization	Guidelines document type
American Academy of Dental Sleep Medicine (AADSM)	Practice Guidelines<br>Consensus positions
American Academy of Oral Medicine (AAOM)	Clinical Practice Statements
American Academy of Pediatric Dentistry (AAPD)	Oral Health Policies<br>Recommendations: Best Practices<br>Recommendations: Clinical Practice Guidelines
American Academy of Pediatrics (AAP)	Clinical report<br>Policy Statement
American Academy of Periodontology (AAP)	Best evidence consensus statement
American Dental Association (ADA)	Clinical Guidelines
American Society of Clinical Oncology (ASCO)	Guidelines
Asociación Latinoamericana de Odontopediatría (ALOP)	Guías [Guidelines]
Australian Dental Association (ADA)	Guidelines<br>Policy Statement
Baltic Osseointegration Academy (BOA)	Consensus statements
Centers for Disease Control and Prevention (CDC)	Guidelines and statements
Chinese Association of Stomatology	指南, 标准 [Guidelines, Standards]
Dutch Association of Orthodontists (NVvO)	Adviezen [Advice]
European Academy of Paediatric Dentistry (EAPD)	Policy document: Best clinical practice guidance
European Association for Osseointegration (EAO)	Consensus statements
European Federation of Conservative Dentistry (EFCD)	Guidelines and Recommendations
European Federation of Periodontology (EFP)	Clinical practice guidelines
European Organisation for Caries Research (ORCA)	Web summaries/Consensus
European Society for Endodontology (ESE)	Position statements
Faculty of Dental Surgery - Royal College of Surgeons England	Clinical Guidelines
FDI World Dental Federation (FDI)	Policy statement
German Society for Dental, Oral and Maxillofacial Medicine e. V -- German - Working Group of Scientific Medical Societies- (AWMF)	S3 - Evidenz- und Konsensbasierte Leitlinien<br>[evidence- and consensus-based guideline]<br>S2e - evidenzbasiert leitlinie<br>[evidence-based guideline]<br>S2k - konsensbasierte leitlinie<br>[consensus-based guidelines]<br>S1 - Handlungsempfehlung von Experten<br>[expert recommendation]
Instituto de Evaluación de Tecnologías en Salud e Investigación (IETSI) del Seguro Social del Perú (EsSalud)	Guias de práctica clínica [Clinical practice guidelines]
Instituto Mexicano del Seguro Social - Centro Nacional de Excelencia Tecnológica - México	Guías de práctica clínica [Clinical practice guideline]
International Association of Dental Traumatology (IADT)	Guidelines
International Association of Paediatric Dentistry (IAPD)	Consensus Recommendations
International Caries Consensus Collaboration (ICCC)	Consensus recommendation
Ivory Cross (Dutch Association for Oral Health)	Advies [Advice]
Japanese Society for Temporomandibular Joint	診療ガイドライン [Clinical practice guideline]
Kennisinstituut Mondzorg (KIMO) - Dutch Institute of Expertise for Oral Healthcare / Royal Dutch Society for the Promotion of Dentistry (KNMT)	Richtlijnen [Guidelines]

Ministry of Health - Brazil	Diretrizes [Guidelines]
Ministry of Health - Chile	Guías clínicas [Clinical Guidelines]<br>Recomendaciones y Normas [Recommendations and Standards]
Ministry of Health - Ecuador	Guía de práctica clínica [Clinical practice guideline]
Ministry of Health - Italy	National guidelines/ Clinical recommendations
Ministry of Health - Malaysia (Kementerian Kesihatan)	Clinical practice guidelines
Ministry of Health - Manatū Hauora - New Zealand (NZ)	Guides and standards
Ministry of Health - New South Wales (NSW)	Guidelines
Ministry of Health - Perú	Guía de práctica clínica [Clinical practice guideline]
National Institute for Health and Care Excellence (NICE)	Guidelines and Quality standards
New Zealand Dental Association (NZDA)	Position statement
Sociedad Española de Epidemiología y Salud Pública Oral (SESPO)	Protocolos [Protocols]
Sociedad Española de Periodoncia y Osteointegración (SEPA)	Guía de practica clínica/Documentos de consenso [Clinical practice guideline/Consensus document]
Société Française de Stomatologie, Chirurgie Maxillo-Faciale et Chirurgie Orale (SFSCMFCO)	Recommandations pour la pratique clinique [Recommandations for clinical practice]
The Belgian Health Care Knowledge Centre (KCE)	Good Clinical Practice
The Scottish Dental Clinical Effectiveness Programme (SDCEP)	Guidance
U.S. Preventive Services Task Force	Recommendation statement
