# Supplementary material for: Evidence-informed guidelines in oral health: insights from a systematic survey
Source: BMC Oral Health. 2024 Jun 27;24:746. doi: 10.1186/s12903-024-04445-w (PMC11212404; doi:10.1186/s12903-024-04445-w)
Supplement: Supplementary file 3 — Supplementary Material 3 [file 12903_2024_4445_MOESM3_ESM.docx]

**Appendix 3. Characteristics of oral health care and oral public health-related guideline documents**

|  | **Implementation level** (n,%) | **Guideline intended users** (n,%) | **Panel composition** (n,%) | **COI policy management*** (n,%) | **Funding source** (n,%) | **Actionable statements** (n,%) |
| --- | --- | --- | --- | --- | --- | --- |
| **Guidelines containing oral health care recommendations**  **(37 organizations, 42 documents types)** | 0 (0%)  Subnational  33 (78%)  National  4 (10%) Regional  5 (12%)  Global | 27 (64%) Health care professionals  11 (26%) Multistakeholder  4 (10%) Not reported | 39/42 (93%) Clinicians | 16 (38%)  Yes  26 (62%)  No or unclear | 27 (64%)  Yes  8 (19%)  Government  2 (5%) Industry  17 (40%)  Professional association or scientific society  15 (36%)  No or unclear | 16/42 (38%)  Formal recommendations |
|  |  |  | 19/42 (45%) Methodologists |  |  |  |
|  |  |  | 7/42 (17%) Policy-makers |  |  |  |
|  |  |  | 8/42 (19%)  Patient partners |  |  |  |
|  |  |  | 2/42 (5%)  Not reported |  |  |  |
| **Guidelines containing oral public health-related recommendations   (n=7 organizations, 7 documents types)** | 1 (14%)  Subnational  6 (86%)  National  0 (0%) Regional  0 (0%)  Global | 4 (57%) Health care professionals  1 (14%) Multistakeholder  2 (29%) Not reported | 5/7 (71%)  Clinicians | 1 (14%)  Yes  6 (86%)  No or unclear | 4 (57%)  Yes  3 (43%)  Government  0 (0%) Industry  1 (14%)  Professional association or scientific society  3 (43%)  No or unclear | 1/7 (14%)  Formal recommendations |
|  |  |  | 2/7 (29%) Methodologists |  |  |  |
|  |  |  | 4/7 (57%) Policy-makers |  |  |  |
|  |  |  | 1/7 (14%)  Patient partners |  |  |  |
|  |  |  | 2/7 (29%)  Not reported |  |  |  |
| **Guidelines containing both oral health care and oral public health-related recommendations   (n=6 organizations, 6 documents types)** | 0 (0%)  Subnational  5 (83%)  National  0 (0%) Regional  1 (17%)  Global | 3 (50%) Health care professionals  2 (34%) Multistakeholder  1 (17%) Not reported | 4/6 (67%)  Clinicians | 1 (17%)  Yes  5 (83%)  No or unclear | 2 (34%)  Yes  1 (17%)  Government  0 (0%) Industry  1 (17%)  Professional association or scientific society   4 (66%)  No or unclear | 2/6 (34%)  Formal recommendations |
|  |  |  | 1/6 (17%) Methodologists |  |  |  |
|  |  |  | 2/6 (33%) Policy-makers |  |  |  |
|  |  |  | 1/6 (17%)  Patient partners |  |  |  |
|  |  |  | 2/6 (33%)  Not reported |  |  |  |
| **Guidelines containing clinical practice recommendations or oral public health-related recommendations (total)  (n=46 organizations, 55 documents types)** | 1 (2%)  Subnational  44 (80%)  National  4 (7%) Regional  6 (11%)  Global | 34 (62%) Healthcare professionals  14 (25%) Multistakeholder  7 (13%) Not reported | 48/55 (87%)  Clinicians | 18 (33%)  Yes  37 (67%)  No or unclear | 33 (60%)  Yes  12 (22%)  Government  2 (4%) Industry  19 (34%)  Professional association or scientific society  22 (40%)  No or unclear | 19/55 (35%)  Formal recommendations |
|  |  |  | 22/55 (40%) Methodologists |  |  |  |
|  |  |  | 13/55 (24%) Policy-makers |  |  |  |
|  |  |  | 10/55 (18%)  Patient partners |  |  |  |
|  |  |  | 6/55 (11%)  Not reported |  |  |  |
| *Conflict of Interest (COI) | | | | | | |
